# Intradural Extramedullary Spinal Cord Metastasis of Breast Cancer in a Male: A Case Report

**DOI:** 10.1002/cnr2.70382

**Published:** 2025-10-29

**Authors:** Hadi Mohammed Abdullah, Naa Adzoa Adzeley Boi‐Dsane, George Wepeba, Thomas Dakurah

**Affiliations:** ^1^ Department of Neurosurgery Korle‐Bu Teaching Hospital Accra Ghana; ^2^ Department of Epidemiology The Noguchi Memorial Institute for Medical Research, University of Ghana Accra Ghana; ^3^ Department of Surgery University of Ghana Medical School Accra Ghana

**Keywords:** breast cancer, intradural extramedullary, Korle‐Bu teaching hospital, metastasis, spinal cord

## Abstract

**Introduction:**

Breast cancer in males is rare, accounting for just 0.5% to 1% according to World Health Organization data. This is the first reported case of IESCM from breast cancer in an African male, which makes it noteworthy. Furthermore, unlike previously reported cases in females, this case involved L1–L2 metastasis with sphincter dysfunction and a subsequent relapse leading to mortality, thereby expanding the documented spectrum of IESCM presentations and outcomes.

**Case Presentation:**

This is a case of a 77‐year‐old male with invasive ductal carcinoma of the left breast and intradural extramedullary spinal cord metastasis diagnosed via Magnetic Resonance Imaging after presenting with neurological symptoms 4 years post‐mastectomy. He eventually passed away following a right Deep Venous Thrombosis, which led to bilateral pulmonary embolism after his second relapse.

**Conclusion:**

Late presentation most likely contributed to the worsening of symptoms and poor prognosis. This report overstates the importance of prompt access to healthcare and the essence of thorough investigations, especially in breast cancer, where neurological symptoms may point to a metastatic diagnosis.

## 1 Background

According to the World Health Organization, breast cancer in males is rare, accounting for just 0.5% to 1% [1]. In Ghana, the incidence is relatively higher, as it accounts for 2.9% of all breast cancers [2]. About 21% of breast cancers metastasise to the spinal cord [3]. Intradural extramedullary spinal cord metastasis (IESCM) of breast cancer is rare, accounting for less than 5% [4].

To the best of our knowledge, there is no reported case of males with IESCM as a result of breast cancer in an African male. We therefore report a case of a 77‐year‐old male with IESCM.

## 2 Case Summary

This is a case of a 77‐year‐old male with invasive ductal carcinoma (ER and PR positive, HER2 negative) at stage T2N2Mx who had a left mastectomy with axillary lymph node clearance in 2012 at Korle‐Bu Teaching Hospital. He then completed adjuvant chemoradiotherapy 2 years later (in 2014) at the same facility.

The adjuvant chemotherapy included Doxorubicin and cyclophosphamide for 6 cycles, followed by 4 cycles of Paclitaxel. For radiotherapy: External beam therapy standard dose fractionation of 50 Gy in 25 fractions.

The right breast was grossly normal following examinations and investigations. He followed this treatment with oral tamoxifen for 2 years. He reported worsening progressive back pain and paraparesis in March 2018 after the initiation of oral tamoxifen therapy at the same healthcare facility.

The patient had been well since then, until 4 years post‐mastectomy, when he developed progressive weakness in the lower limbs with both bowel and bladder dysfunction. He reported severe progressive back pain radiating to the left thigh. Subsequently, within the same year, he also experienced paraparesis for 6 months and urinary incontinence for a month. There was no family history of any form of cancer and no other positive risk factors for breast cancer aside from his previous history. There was no new breast lesion on physical examination. The power in the upper limbs was 5/5, but 4/5 in both lower limbs, with normal reflexes in all areas.

Laboratory results of metastatic workup conducted are as follows: PSA < 4 ng/mL (ref: < 4 ng/mL), ALT < 55 U/L (7–55 U/L), AST < 48 U/L (8–48 U/L), ALP < 147 U/L (44–147 U/L), Bilirubin < 1.2 mg/dL (0.1–1.2 mg/dL), Creatinine < 1.3 mg/dL (0.6–1.3 mg/dL).

Neurological assessment intact (cranial nerves, cerebellar signs, and sensory function). Chest X‐ray and abdominopelvic ultrasound showed no pulmonary/abdominal metastases. Central Nervous System imaging revealed no intracranial lesions.

**FIGURE 1:**
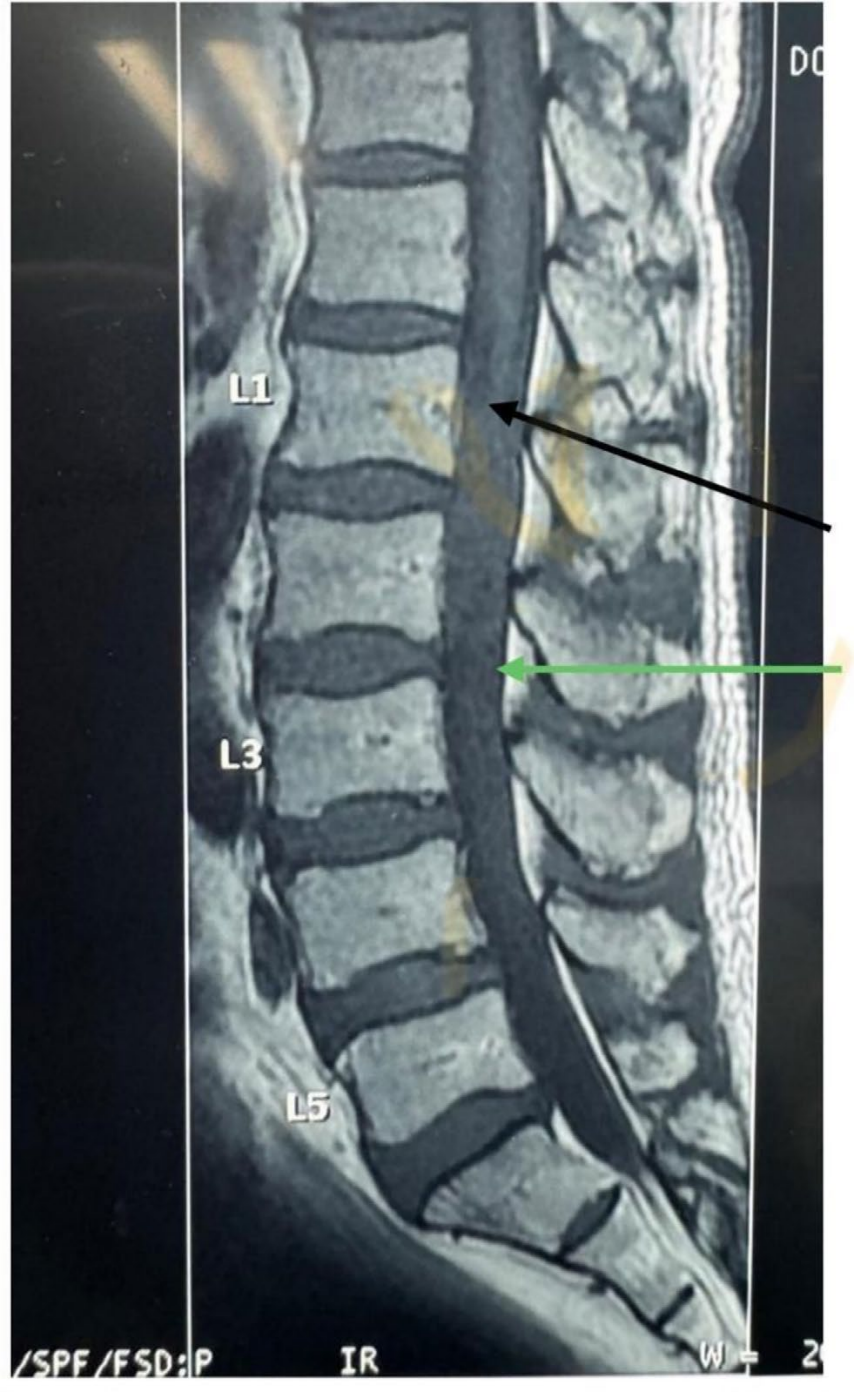
Pre‐operative magnetic resonance imaging T1 weighted image shows a 4.33 cm craniocaudal, 1.60 cm transverse, and 1.0 cm AP diameter, well‐defined, sharply marginated oval shape, isointense intradural extramedullary mass extending from L1 to L2. It fills the spinal canal. The arrows show an isointense intramedullary spinal cord tumour. The black arrow shows the proximal extent/border while the green arrow shows the caudal extent.

**FIGURE 2:**
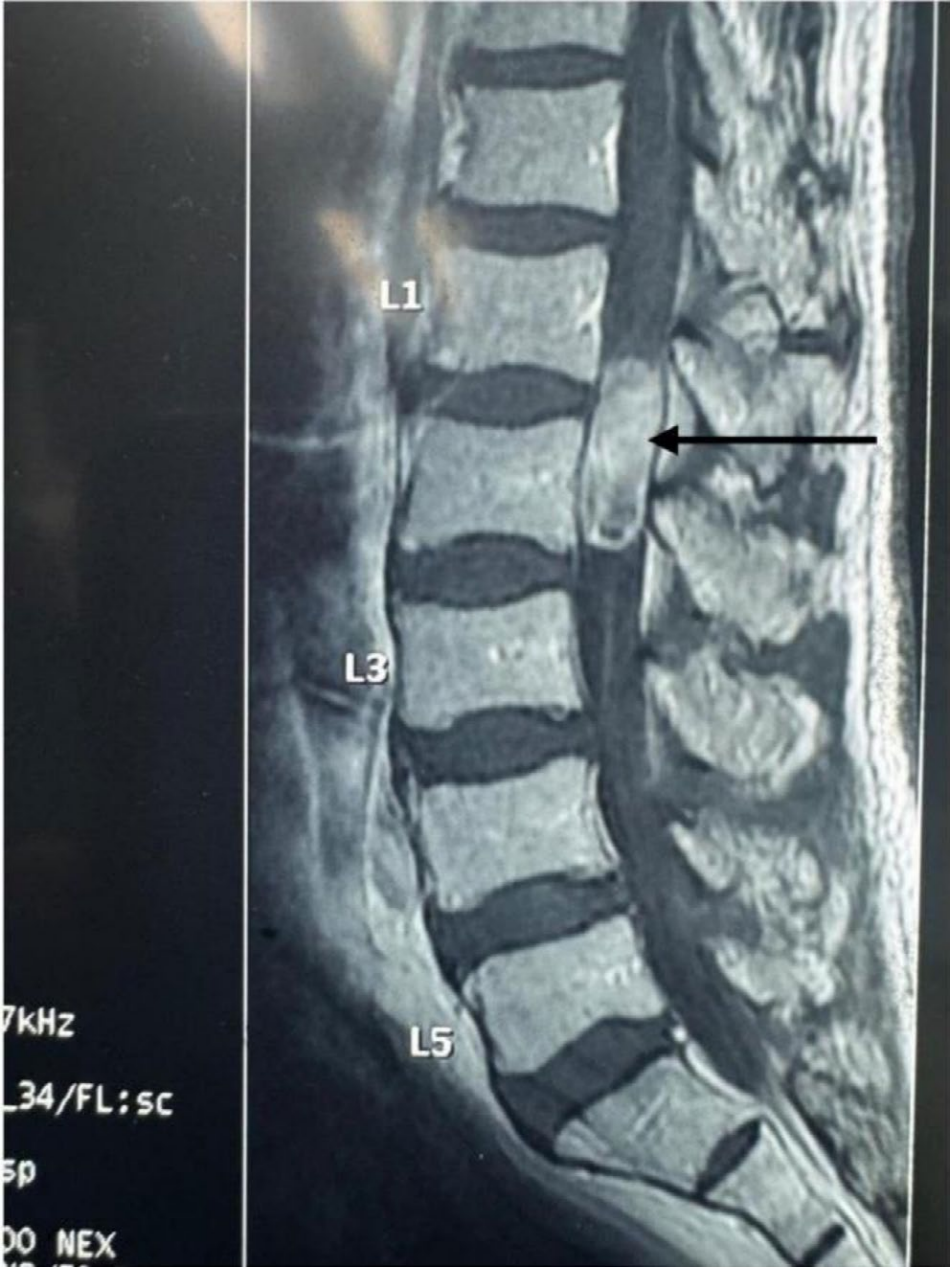
Pre‐operative T1‐weighted image of the spine. The black arrow points to heterogenous but intensely enhancing intramedullary tumour on T1WI.

**FIGURE 3:**
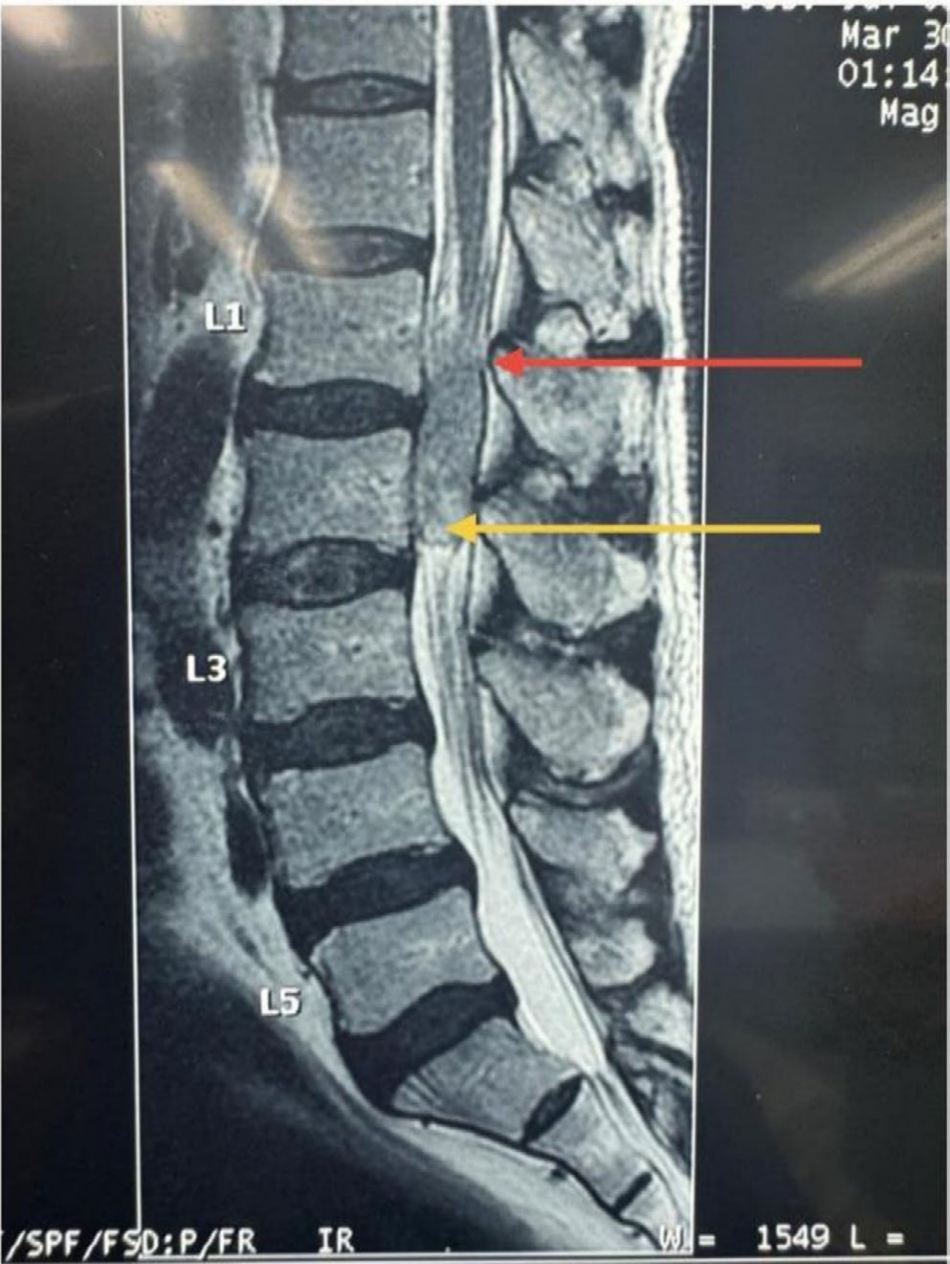
Pre‐operative T2 weighted image, which shows a slightly hyperintense, well‐defined intradural extramedullary lesion that occupies the spinal canal. The tumour extends to the conus medullaris and the cauda equina with the resultant compression of the nerve roots. The red arrow is pointing to the conus extent of the lesion and yellow arrow is pointing to the cauda equina extent. The lesion is slightly hyperintense on T2WI.

The Magnetic Resonance Imaging (MRI) scan showed involvement of L1–L2, as seen in Figures 1, 2, 3. There was a fairly defined 4.33 cm craniocaudal, 1.60 cm transverse, and 1.0 cm AP diameter lesion, a sharply marginated oval‐shaped intradural extramedullary tumour that fills the spinal canal, compressing the conus with flattening of the nerve roots. No gross features of cord invasion and no attachment to the dura were seen. The abdominopelvic ultrasonography scan, chest, pelvic, and spinal cord X‐rays showed no tumour or metastatic lesions. Intradural extramedullary tumour for which he had L1–L2 laminectomy and gross total excision. The tumour was reddish‐cream, friable and suckable, attached to some nerve roots at the conus and the nerve roots of the cauda equina. But not attached to the dura mater. It was not intramedullary; it was all extramedullary. Areas of vascularization are from branches of the spinal artery. The residual tumour was located dorsally. It was in the distal midline.

**FIGURE 4:**
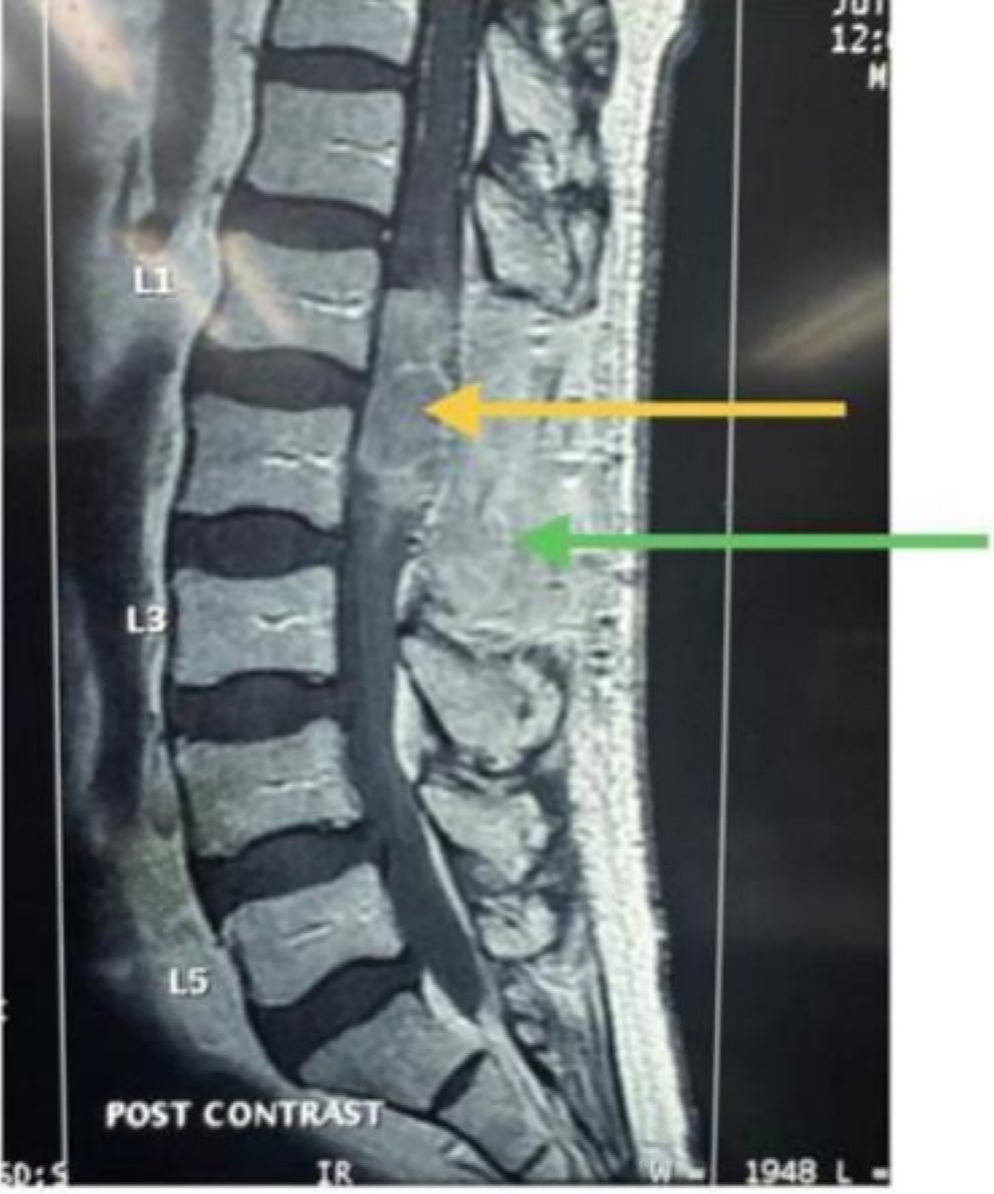
Post contrast T1 weighted image shows a 6‐month post‐laminectomy and excision MRI with contrast, showing recurrence of the tumour. The tumour extends beyond the lower endplate of L1 as seen before surgery. This shows an increase in the size of the tumour. There is an extensive contrast‐enhancing scar tissue involving the laminectomy defect. Yellow arrow shows an enhancing lesion extending from conus to cauda equina. Green arrow shows an L1–L2 lamina defect with extensive enhancing scar within the defect.

The histology revealed metastatic invasive carcinoma likely of breast origin. Immunohistochemistry showed that there was triple‐negative breast cancer with a Ki67 of 46%. He improved neurologically post‐operatively and started mobilising with the aid of a Zimmer frame. He, however, had a relapse within 6 months, and an MRI was done as shown in Figure 4, showing recurrence of the tumour post‐surgery. There was also extensive scar tissue, which was also enhanced with contrast. The right breast had no palpable lesion. The mammogram done was negative for tumour in the right breast. While preparing for adjuvant chemoradiation, he developed right lower limb deep vein thrombosis (DVT) and died from massive bilateral pulmonary embolism.

There were diagnostic challenges in this case, with a major one being the high cost of an MRI scan. Due to financial constraints, a comparative MRI scan was not available to allow for further evaluation of the resection margin for residuals. Furthermore, significant details, including test results, imaging, and previous management details during the initial management, were lost in transition from the peripheral facility to the tertiary facility.

Table 1 summarises the timeline of events for this case from diagnosis to management to death.

**TABLE 1:** Shows the timeline of the events from diagnosis to management till death.

Year	Event
2012	Diagnosed with invasive ductal carcinoma at stage T2N2Mx (ER/PR positive, HER2 negative). Underwent left mastectomy with axillary lymph node clearance.
2014	Completed adjuvant chemoradiotherapy. Started oral tamoxifen therapy.
2016	Completed 2 years of tamoxifen treatment
March 2018	Developed worsening back pain and paraplegia. Diagnosis of L1–L2 intradural extramedullary tumour by MRI, and underwent L1–L2 laminectomy and gross total excision of the tumour. Histology confirmed metastatic invasive carcinoma of breast origin. The patient had postoperative neurological improvement with mobilisation using a Zimmer frame.
September 2018	Experienced relapse within 6 months post‐surgery. Developed right DVT while preparing for adjuvant chemoradiation, resulting in bilateral pulmonary embolism leading to the patient's demise

## 3 Discussion

According to data published by the Centres for Disease Control and Prevention (CDC), male breast cancer occurs in approximately 1.3% of all breast cancers in the United States of America (USA), with black males being the most affected (about higher incidence and mortality rates) [5], which is relevant especially in an African setting due to similar characteristics. In Ghana, the rate of male breast cancer is more than double that of the aforementioned CDC data in the USA, culminating in about 2.9% in a 2014 study published by Quayson et al. The same study also reports higher rates of male breast cancer within West Africa, ranging from 2.0% to 3.8% [2]. Male breast cancer usually occurs in the elderly at about age 71 [6], which is in line with the age of the man reported to have breast cancer in our case report. The invasive ductal carcinoma type is most common (as was the same in our case) in males (making up 90%) worldwide, as well as in Accra [3, 7], where the subject of the study is located. About 90% of males with breast cancer are Estrogen Receptor (ER positive), and 81% are Progesterone Receptor (PR positive) [8]. Therefore, it was not surprising that the patient in our case report also turned out to have ER and PR positive breast cancer initially, which tends to have a better prognosis as opposed to triple‐negative breast cancer. However, his subsequent triple‐negative status most likely compounded his poor prognosis when he had a relapse.

Since the patient was being managed in another facility prior to presentation to the referral site, some details of his management from presentation are unclear. The common symptom at presentation is a painless unilateral breast mass [6]. Similarly, this was seen in our case, where the right breast was normal. Occasionally, there may be nipple discharge, pain, ulceration, and/or retraction with gynecomastia, posing a diagnostic challenge [6, 7]. This information was, however, not available in our case report since the patient presented post‐mastectomy. It is worth noting that the patient presented when the breast cancer was in an advanced metastatic state, as is often seen in literature, where males are diagnosed with late‐stage breast cancer at presentation [7]. In our case, due to spinal metastasis at late presentation, the patient started developing bladder and bowel incontinence associated with paraparesis.

Intradural extramedullary spinal cord metastasis (IESCM) of breast cancer accounts for less than 5% [4]. This was also a case of intradural extramedullary spinal cord metastasis, which adds to the rarity.

A systematic review by Scalia et al. in 2023 revealed that only 1 male patient had intramedullary spinal cord metastasis from breast cancer out of 123 patients from 44 studies, with none of the metastases being extramedullary [9]. However, there was a reported case in 2022 of intradural extramedullary metastasis secondary to primary breast cancer, but this was in a 60‐year‐old female [10]. The patient under discussion was male, older, and had no brachial plexus involvement as opposed to that of the aforementioned case. Similarly, there has been another reported case (also in a younger woman) a year later, who had T12–L1 involvement due to the metastasis from breast cancer [11] as opposed to L1–L2 involvement in this case. So far, none of the reported cases in the literature have been male, which adds to the uniqueness of this case. A systematic review conducted by Palmisciano et al. found that intradural extramedullary metastasis usually occurs in the elderly, with lung, kidney, and breast being the most common primary tumours in descending order, with a median time of 18 months till metastasis [12] but the patient did not have symptoms until 4 years later in our case. The aforementioned study also corroborated our findings of paraparesis and sphincter dysfunction being a common symptom at presentation, with the imaging modality of choice being MRI, as was done in our case, as well as 93.3% of their cases reviewed [12]. Decompressive laminectomy with tumour resection was the treatment modality of choice in all cases, with more than half being partial [12]. However, a total excision with a laminectomy was done in our case due to the severity of symptoms and extent of metastasis. About 70.8% of patients with IESCM have symptomatic relief post‐treatment [12]. The mortality rate for metastatic IESCM cases is usually 50% with 6.7 months being the median survival rate [10] as was seen in our case, when the patient died 6 months post‐operatively after another relapse. Upon conducting an extensive literature review, to the best of our knowledge, no published case of IESCM from breast cancer was found in an African male. The only case report on IESCM from breast cancer was reported in a female known to have brachial plexitis who also presented with limb weakness but no bladder or bowel dysfunction [11]. The left breast was also affected in the case report by Dandekar et al., with limb weakness being present as was in our case; however, the distinguishing factor was the functionality of the sphincter and presence of brachial plexitis, believed to have influenced the perineural spread in their case. The metastasis in their case affected C5–T1 [11], but the involvement of the spinal cord was at a lower level at L1/L2 in our case. The patient was followed up till she was started on hormonal therapy [11]; however, in our case, the patient started experiencing metastatic symptoms 2 years post‐hormonal therapy and eventually died from bilateral pulmonary embolism following right lower limb DVT. Pulmonary embolism comes second to cancer as the cause of death in patients with cancer [10].

Management options for breast cancer are dependent on the stage of the disease as well as prognosis. The patient was initially managed by left mastectomy, which is the mainstay in stage T1 and T2, but is usually a simple type. Furthermore, in T3 tumours or advanced cases, radiotherapy is also done, especially where there are more than three positive lymph nodes or positive tumour margins. The patient is also required to be put on tamoxifen for 5 years, especially in ER‐positive cancers, which are mostly metastatic [9], as seen in our case, but this patient was on the hormonal therapy for just 2 years before his relapse. This patient had a combination of mastectomy, with tamoxifen therapy as well as adjuvant radiotherapy, most likely owing to the stage and aggressiveness of the cancer.

## 4 Conclusion

This is a report of a rare case of intradural extramedullary metastasis from left breast cancer in a male with two relapses, which ended in mortality. The late presentation most likely contributed to the worsening of symptoms and poor prognosis. The essence of this report is to alert other clinicians to look beyond the bony spine, which is one of the areas of metastasis for breast cancer, and increase surveillance of the spinal cord for metastasis. This report contributes to global literature by documenting the first case of IESCM from breast cancer in an African male, with distinct clinical features compared to previously reported female cases. Furthermore, this report overstates the importance of prompt access to healthcare and the essence of thorough investigations, especially in breast cancer, where neurological symptoms may point to a metastatic diagnosis.

## Author Contributions

**Hadi Mohammed Abdullah:** conceptualization, design, writing – review and editing, final approval. **Naa Adzoa Adzeley Boi‐Dsane:** design, writing – original draft, review and editing. **George Wepeba:** review and editing, final approval, supervision. **Thomas Dakurah:** review and editing supervision. All authors are accountable for this manuscript.

## Consent

Informed consent was obtained from the patient and relatives before he died for both surgery and publication of this case report.

## Conflicts of Interest

The authors declare no conflicts of interest.

## Data Availability

The data that support the findings of this study are available from the corresponding author upon reasonable request.
